# Male Patients With Dilated Cardiomyopathy Exhibiting a Higher Heart Rate Acceleration Capacity or a Lower Deceleration Capacity Are at Higher Risk of Cardiac Death

**DOI:** 10.3389/fphys.2018.01774

**Published:** 2018-12-07

**Authors:** Yichen Yang, Fengyan Wang, Cao Zou, Hongkai Dong, Xingmei Huang, Bingyuan Zhou, Xun Li, Xiangjun Yang

**Affiliations:** ^1^Department of Cardiology, The First Affiliated Hospital of Soochow University, Suzhou, China; ^2^Department of Cardiology, People’s Hospital of Rizhao, Rizhao, China; ^3^Department of Cardiology, Yuncheng Central Hospital, Yuncheng, China; ^4^Department of Electrocardiography, The First Affiliated Hospital of Soochow University, Suzhou, China; ^5^Department of Echocardiography, The First Affiliated Hospital of Soochow University, Suzhou, China

**Keywords:** dilated cardiomyopathy, cardiac autonomic regulation, electrophysiology, heart rate acceleration capacity, heart rate deceleration capacity

## Abstract

The effects of dilated cardiomyopathy (DCM) on cardiac autonomic regulation and electrophysiology, and the consequences of such changes, remain unclear. We evaluated the associations between heart rate acceleration capacity (AC) and deceleration capacity (DC), heart structural and functional changes, and cardiac death in 202 healthy controls and 100 DCM patients. The DC was lower and the AC was higher in DCM patients (both males and females). Multivariable, linear, logistic regression analyses revealed that in males, age was positively associated with AC in healthy controls (*N* = 85); the left atrial diameter (LAD) was positively and the left ventricular ejection fraction (LVEF) was negatively associated with AC in DCM patients (*N* = 65); age was negatively associated with DC in healthy controls (*N* = 85); and the LAD was negatively and the LVEF was positively associated with DC in DCM patients (*N* = 65). In females, only age was associated with either AC or DC in healthy controls (*N* = 117). Kaplan–Meier analysis revealed that male DCM patients with greater LADs (≥46.5 mm) (long-rank chi-squared value = 11.1, *P* = 0.001), an elevated AC (≥-4.75 ms) (log-rank chi-squared value = 6.8, *P* = 0.009), and a lower DC (≤4.72 ms) (log-rank chi-squared value = 9.1, *P* = 0.003) were at higher risk of cardiac death within 60 months of follow-up. In conclusion, in males, DCM significantly affected both the AC and DC; a higher AC or a lower DC increased the risk of cardiac death.

## Introduction

Dilated cardiomyopathy (DCM) is a progressive disease of the heart muscle characterized by the presence of left ventricular dilatation and systolic dysfunction (Elliott et al., 2008). DCM is attributable to genetic variations, alcohol and drug abuse, certain toxins, pregnancy, certain infections, and metabolic and endocrine disturbances (Weintraub et al., 2017). DCM is one of the most common causes of heart failure in young patients, and is a major cause of sudden cardiac death (Elliott et al., 2008; Japp et al., 2016; Weintraub et al., 2017). The patho-anatomical characteristics of DCM, such as heart failure, valve disease and heart blood clots, have been well studied (Elliott et al., 2008; Japp et al., 2016; Weintraub et al., 2017). A dilated heart does not pump blood effectively, and enlargement of heart chambers compromises valve function (Elliott et al., 2008; Japp et al., 2016; Weintraub et al., 2017). However, the effects of DCM on cardiac autonomic regulation and electrophysiology, and the consequences of such changes, remain unclear.

In clinical practice, some DCM patients had concomitant supraventricular arrhythmias or conduction disease; and such electric instability could not be explained reasonably by existing pathophysiological principles of DCM (Lakdawala and Givertz, 2010; Lakdawala et al., 2013). The heart is richly innervated by sympathetic and parasympathetic (vagal) nerves that differ in the neurotransmitters employed and exert stimulatory or inhibitory effects on target cells via adrenergic or muscarinic receptors (Florea and Cohn, 2014). The normal electrophysiological activities of the sinoatrial and atrioventricular nodes are modulated by paired sympathetic and vagal nerves, respectively (Colucci et al., 1986; Florea and Cohn, 2014). The ventricular myocardium is controlled by a branch of the sympathetic nerve (Bristow et al., 1986; Colucci et al., 1986; Florea and Cohn, 2014). Increased heart sympathetic adrenergic activity combined with a reduction in vagal activity trigger pathophysiological neurohormonal changes developing secondary to DCM (Weintraub et al., 2017). It is important to understand how such changes in sympathetic and parasympathetic innervation occurred and what is the consequence of these modulation changes in the sinoatrial node, atrioventricular node and the ventricular myocardium of patients with DCM.

The phase-rectified signal averaging (PRSA) technique detects and quantifies quasi-periodic oscillations that masked by non-periodic components, artifacts, and/or ectopic beats (Kantelhardt et al., 2007). Using the PRSA technique, we could calculate heart rate acceleration capacity (AC) and deceleration capacity (DC) based on 24-h ambulatory electrocardiograms records. Researches revealed that the AC and DC are associated with mortality after myocardial infarction and heart failure stages in DCM patients with high predicting performance (Bauer et al., 2006; Hu et al., 2016; Zou et al., 2016). Assuming that AC and DC could reflect the sympathetic and parasympathetic nervous modulation in heart, we could learn the pathophysiological changes of autonomic nervous regulation in DCM from a new angle. In this report, the AC and DC of healthy controls and DCM patients were measured; the association between AC/DC and risk of cardiac death were evaluated by a longitudinal study.

## Materials and Methods

### Ethical Issues

The review board of the First Affiliated Hospital of Soochow University approved this study, which adhered to all principles of the revised Declaration of Helsinki. Written informed consent (for the planned procedures and use of medical data) was obtained from all patients as dictated by the guidelines of the Chinese National Ethics Regulation Committee. All participants and their immediate relatives, caregivers, or legal guardians were told that they could withdraw consent at any time.

### Subjects

**FIGURE 1:**
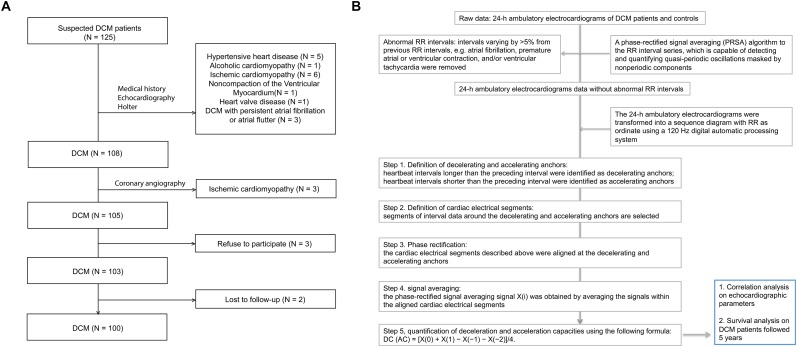
Flowchart of patient selection and a schematic diagram of AC/DC calculation. DCM, dilated cardiomyopathy. **(A)** Flowchart of patient selection. **(B)** A schematic diagram of AC/DC calculation.

Dilated cardiomyopathy patients treated between January 2013 and June 2016 were enrolled. DCM was diagnosed when the ejection fraction was < 0.45 and/or a fractional shortening < 25% was apparent, combined with a left ventricular end-diastolic dimension (LVEDD) > 112% of the predicted value after correction for age and body surface area (Elliott, 2000). Exclusion criteria were: systemic hypertension (>160/100 mmHg); coronary artery disease (>50% in one or more major branches); chronic alcohol consumption (>40 g/day in females; >80 g/day in males); any systemic disease known to cause DCM; any pericardial disease; congenital heart disease; and/or a cor pulmonale (Elliott, 2000). A total of 100 DCM patients (65 males and 35 females) were finally enrolled (Figure 1A). Heart failure (HF) classification followed the recommendations of the American College of Cardiology Foundation, and the criteria of the American Heart Association were used to diagnose HF stage (Yancy et al., 2013).

A total of 202 healthy controls who underwent physical examinations in our hospital served as normal controls (males, 86; females, 116, age 54 ± 13.2 years). All controls were confirmed to not have DCM, atrial fibrillation, atrial flutter, pacemakers, sick sinus syndrome, an atrioventricular block, hypertension, coronary heart disease, even limited cardiomyopathy, pulmonary heart disease, myocarditis, valvular heart disease, congenital heart disease, any neuromuscular disease, any liver or kidney dysfunction, and diabetes.

### Calculation of AC and DC Using 24-h Ambulatory Electrocardiograms

As we reported previously (Figure 1B) (Zou et al., 2016), a PRSA algorithm detects and quantifies quasi-periodic oscillations masked by non-periodic components, artifacts, and ectopic beats (Kantelhardt et al., 2007). We used this algorithm to analyze 24-h ambulatory electrocardiograms. The DC and AC were computed at a timescale (T) of unity and a wavelet scale (s) of 2. Abnormal RR intervals (intervals varying by >5% from previous RR intervals (in terms of atrial fibrillation, premature atrial or ventricular contraction, and/or ventricular tachycardia) were removed to minimize errors caused by artifacts and variations in ectopic rhythm. Briefly, in step 1 (definition of decelerating and accelerating anchors), heartbeat intervals longer than those of preceding intervals were considered to reflect deceleration and intervals shorter than the preceding intervals were defined as accelerating anchors. In step 2 (definition of cardiac electrical segments), segmental interval data surrounding the decelerating and accelerating anchors were evaluated. In step 3 (phase rectification), the electrical cardiac segments described above were aligned to the decelerating and accelerating anchors. In step 4 (signal averaging), phase-rectified signal averages [X(i) values] were obtained by averaging signals within aligned cardiac electrical segments. In step 5, deceleration and acceleration capacities were quantified using the following formula: DC (AC) = [X(0)+X(1)–X(–1)–X(–2)]/4 (Bauer et al., 2006; Hu et al., 2016; Zou et al., 2016).

### Echocardiography

As previously described in detail (Zou et al., 2016), transthoracic echocardiographic examinations were performed using a Sonos 5500 Ultrasound machine (Philips, Best, Netherlands) fitted with a 2.5-Hz transducer. Parameters measured using the M-mode technique included the left ventricular end-diastolic diameter (LVEDD, normal range 35–56 mm), the left ventricular end-systolic diameter (LVESD, normal range: 20–40 mm), and the left atrial diameter (LAD, normal range 27–40 mm). The left ventricular ejection fraction (LVEF, normal value: >0.45) was measured using the Simpson biplane method. All echocardiographic examinations were performed and data were analyzed by the same experienced echocardiographer who was blinded to clinical data and patient group. Any uncertainties in echocardiographic data were resolved by discussion among senior technicians in the department of echocardiography.

### Statistical Analysis

The distributions of all variables were first assessed by drawing histograms; correlations between variables were then calculated using scatter plots and Pearson’s correlation coefficient analysis. All analyses were performed separately for either gender.

We used three progressive analysis strategies to evaluate associations between AC, DC, and DCM. First, we compared the AC, DC, and echocardiographic indices between DCM patients and healthy controls. The paired Student’s *t*-test was used for comparisons within groups and the non-paired Student’s *t*-test for comparisons between groups; all tests were two-sided. Next, the associations between the AC/DC and echocardiographic indices and age were subjected to univariate linear logistic regression, and factors associated with *P-*values < 0.1 underwent further multivariable linear logistic regression. Pearson’s correlation revealed a close link between AC and DC, and we thus performed linear logistic regression using either AC or DC as a dependent variable. Third, we performed Kaplan–Meier analyses with log-rank testing to calculate survival rates over the 60 months of follow-up and to compare survival rates between groups. The LAD, DC, and AC cutoffs were selected by reference to the areas under the receiver operating characteristic (ROC) curves (AUCs). *P*-values < 0.05 were deemed to reflect statistical significance. All statistical analyses were performed with the aid of SPSS software version 17.0 (SPSS Inc., Chicago, IL, United States).

## Results

### Distributional Differences Between Patients and Controls

In males, the average AC, LAD, LVEDD, and LVESD were significantly higher in DCM patients than controls; conversely, the average DC and LVEF were significantly lower than in controls (Table 1). In females, DCM patients were significant older than controls and, as with males, the average AC, LAD, LVEDD, and LVESD were significantly higher in DCM patients than controls; conversely, the average DC and LVEF were significantly lower in DCM patients (Table 1). In summary, the echocardiographic data confirmed the pathophysiological changes associated with DCM; both the AC and DC were significantly affected.

**Table 1:** Differences between DCM patients and controls.

Indices	Males (*N* = 150)			Females (*N* = 152)		
	Controls (*N* = 85)	DCM patients (*N* = 65)	*P*	Controls (*N* = 117)	DCM patients (*N* = 35)	*P*
***Demographic data***					
Age, years	53.4 11.9	52.8 14.1	0.775	54.7 14.1	62.8 8.7*	0.002
***Deceleration and acceleration capacity***					
AC, ms	-7.9 2.3	-5.0 2.4	<0.001	-7.5 1.8	-4.1 1.4	<0.001
DC, ms	7.6 2.0	4.6 2.3	<0.001	7.1 1.6	3.9 1.3	<0.001
***Echocardiography***					
LAD, mm	38.4 5.9	48.0 10.0	<0.001	37.3 5.6	47.6 7.5	<0.001
LVEDD, mm	49.1 5.5	71.2 8.9	<0.001	48.4 4.2	67.8 7.3	<0.001
LVESD, mm	31.2 6.2	59.6 10.3	<0.001	30.1 3.8	57.9 8.9	<0.001
LVEF, %	65.6 7.8	30.3 7.1	<0.001	67.2 6.1	30.5 8.0	<0.001

*Data are presented as means ± standard deviations (SDs). Differences between groups were compared using Student’s *t*-test. DCM, dilated cardiomyopathy; AC, heart rate acceleration capacity; DC, heart rate deceleration capacity; LAD, left atrial diameter; LVEDD, left ventricular end-diastolic diameter; LVESD, left ventricular end-systolic diameter; LVEF, left ventricular ejection fraction. ^∗^, female DCM patients were significantly older than male DCM patients (*P* < 0.001)*.

### DCM Affected the Correlations Between AC and DC, Age and Echocardiographic Parameters

**FIGURE 2:**
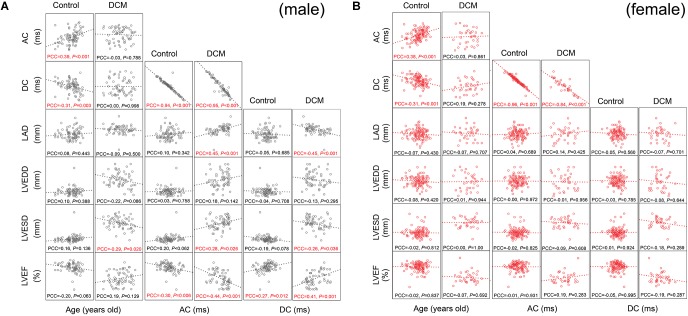
Scatter plot matrix and Pearson’s correlation coefficients among variables. The associations between parameters were presented by scatter plot matrix visually together with Pearson’s correlation coefficients. The scales differed between each subunit of scatter plot matrix, so, we omitted scales of each axes. The accurate quantitative results of linear logistic regression are presented in Tables 2, 3. PCC, Pearson’s correlation coefficient (two-tailed *P*-values are presented). PCC with *P* < 0.05 was labeled in red. Please see Table 1 for a list of other abbreviations. **(A)** Scatter plot matrix and Pearson’s correlation coefficients among variables for males. **(B)** Scatter plot matrix and Pearson’s correlation coefficients among variables for females.

To explore correlations between AC, DC, age, and echocardiographic parameters, we drew scatter plots and calculated Pearson’s correlation coefficients. AC was positively and DC was negatively associated with age in both male and female controls (Figures 2A,B; age column). DCM greatly affected this pattern; in males, DCM abolished the association between AC, DC, and age (Figure 2A; age column); the LVESD was now significantly (negatively) associated with age (Figure 2A; age column). In females, DCM abolished the association between AC, DC, and age, but no new association emerged (Figure 2B; age column).

The AC displayed a very precise negative correlation with the DC in both males and females (Figures 2A,B). Healthy control males exhibited a significant negative association between the AC and LVEF (Figure 2A; AC column). Conversely, no significant association between AC and any echocardiographic parameter was observed in female controls (Figure 2B; AC column). DCM dramatically changed the above associations in males; significant positive associations between AC, LVESD, and LAD were now observed; and the negative slope of the LVEF/AC relationship fell from -0.30 to -0.44 (Figure 2A; AC column). Interestingly, no similar changes were observed in females (Figure 2B; AC column). In conclusion, Pearson’s correlation coefficient analysis revealed extensive associations between the AC and echocardiographic changes in males with DCM.

In male controls, DC was significantly (positively) associated with the LVEF (Figure 2A; DC column). Conversely, no significant association was observed in female controls (Figure 2B; DC column). Again, DCM changed the associations between DC and echocardiographic parameters. DC now became negatively associated with the LAD and LVESD; and the slope of the (positive) association between LVEF and DC increased from 0.27 to 0.41 (Figure 2A; DC column). Interestingly, no similar changes were observed in females (Figure 2B; DC column).

### Factors Associated With AC in Controls and Patients With DCM

We used a univariate linear logistic regression to further assess the association between AC and echocardiographic parameters. Similar to what was noted on Pearson’s correlation coefficient analysis, in male controls, AC increased with age with a *B* value of 0.074 (95% CI, 0.036 ∼ 0.113; *P* < 0.001) and LVEF associated with AC increased with a B value of -8.632 (95% CI, -14.666 ∼ -2.598; *P* = 0.006) (Table 2). In male DCM patients, LVESD was positively associated with the AC with a B value of 0.064 (95% CI, 0.008 ∼ 0.121; *P* = 0.026); the association of LVEF with AC increased with a B value of -14.791 (95% CI, -22.453 ∼-7.129; *P* < 0.001); and the LAD was also positively associated with the AC with a B value of 0.107 (95% CI, 0.053 ∼ 0.161; *P* < 0.001) (Table 2). To adjust for possible interactions among variables, a multivariable linear logistic regression was next performed using variables associated with *P*-values < 0.1 on both univariate regression and Pearson’s correlation coefficient analysis. As shown in Table 2, age was independently (positively) associated with AC in male controls; and the LAD and LVEF were independently (positively and negatively, respectively) associated with AC in male DCM patients.

**Table 2:** Factors associated with AC in controls and patients with DCM.

Indices	Controls (*N* = 202)			DCM patients (*N* = 100)		
	B	95% CI	*P*	B	95% CI	*P*
	*Males (N = 85)*			*Males (N = 65)*		
***Univariate linear logistic regression***			
Age, years	0.074	0.036 ∼ 0.113	<0.001	/	/	/
LAD, mm	/	/	/	0.107	0.053 ∼ 0.161	<0.001
LVEDD, mm	/	/	/	/	/	/
LVESD, mm	0.074	-0.004 ∼ 0152	0.062	0.064	0.008 ∼ 0.121	0.026
LVEF, %	-8.632	-14.666 ∼-2.598	0.006	-14.791	-22.453 ∼-7.129	<0.001
***Multivariable linear logistic regression***			
Age, years	0.065	0.027 ∼ 0.104	0.001	/	/	*/*
LAD, mm	/	/	*/*	0.075	0.018 ∼ 0.133	0.011
LVEDD, mm	/	/	*/*	/	/	*/*
LVESD, mm	/	/	/	/	/	*/*
LVEF, %	/	/	*/*	-9.729	-18.314 ∼-1.144	0.027
	*Females (N = 117)*		*Females (N = 35)*		
***Univariate linear logistic regression***			
Age, years	0.05	0.028 ∼ 0.072	<0.001	/	/	/
LAD, mm	/	/	*/*	/	/	/
LVEDD, mm	/	/	*/*	/	/	/
LVESD, mm	/	/	/	/	/	/
LVEF, %	/	/	/	/	/	/

*B: this is a coefficient indicating a one-unit increase in an independent variable*.

In females, only age was significantly associated with AC on the univariate linear logistic regression analysis, with a *B*-value of 0.050 (95% CI, 0.028 ∼ 0.072; *P* < 0.001); a multivariable linear logistic regression analysis was thus not performed (Table 2).

In summary, age was an independent risk factor associated with AC in both male and female controls, and the LAD and LVEF were independent risk factors associated with AC in male DCM patients.

### Factors Associated With DC in Controls and Patients With DCM

Univariate linear logistic regression analyses revealed that in male controls, age was negatively and LVEF was positively associated with DC with *B* values of -0.05 (95% CI, -0.090 ∼-0.018; *P* = 0.003) and 7.063 (95% CI, 1.587 ∼ -12.539; *P* = 0.012) respectively (Table 3); in DCM patients, the LAD and LVESD were negatively associated with DC with *B* values of -0.102 (95% CI, -0.154 ∼-0.051; *P* < 0.001) and -0.058 (95% CI, -0.112 ∼-0.004; *P* = 0.036) respectively; and LVEF was associated with DC with a *B* value of 13.342 (95% CI, 5.965 ∼ 20.720; *P* = 0.001) (Table 3). Multivariable linear logistic regression revealed that only age was independently associated with DC in healthy controls, with a small change in the *B* value (Table 3); the LAD and LVEF were both independently associated with DC in DCM patients with *B* values of -0.075 (95% CI, -0.130 ∼-0.020; *P* = 0.008) and 8.428 (95% CI, 0.194 ∼ 16.662; *P* = 0.045), respectively (Table 4).

**Table 3:** Factors associated with DC in controls and patients with DCM.

Indices	Controls (*N* = 202)			DCM patients (*N* = 100)		
	B	95% CI	*P*	B	95% CI	*P*
	*Males (N = 85)*			*Males (N = 65)*		
***Univariate linear logistic regression***			
Age, years	-0.054	-0.090 ∼-0.018	0.003	/	/	/
LAD, mm	/	/	/	-0.102	-0.154 ∼-0.051	<0.001
LVEDD, mm	/	/	/	/	/	/
LVESD, mm	-0.063	-0.133 ∼ 0.007	0.078	-0.058	-0.112 ∼– 0.004	0.036
LVEF, %	7.063	1.587 ∼ 12.539	0.012	13.342	5.965 ∼ 20.720	0.001
***Multivariable linear logistic regression***			
Age, years	-0.047	-0.082 ∼-0.011	0.012	/	/	*/*
LAD, mm	/	/	*/*	-0.075	-0.130 ∼-0.020	0.008
LVEDD, mm	/	/	*/*	/	/	*/*
LVESD, mm	/	/	/	/	/	*/*
LVEF, %	/	/	*/*	8.428	0.194 ∼ 16.662	0.045
	*Females (N = 117)*			*Females (N = 35)*		
***Univariate linear logistic regression***			
Age, years	-0.035	-0.055 ∼-0.015	0.001	/	/	/
LAD, mm	/	/	/	/	/	/
LVEDD, mm	/	/	/	/	/	/
LVESD, mm	/	/	/	/	/	/
LVEF, %	/	/	/	/	/	/

*B: this is a coefficient indicating a one-unit increase in an independent variable*.

**Table 4:** Differences between DCM subgroups of those who survived or died.

Indices	Males (*N* = 65)			Females (*N* = 35)		
	Survived (*N* = 45)	Died (*N* = 20)	*P*	Survived (*N* = 30)	Died (*N* = 5)	*P*
***Follow-up***				
Follow-up time, months	29.2 ± 15.9	29.5 ± 14.6	0.946	33.2 ± 15.0	28.2 ± 15.4	0.529

***Demographic data***				
Age, years	50.8 ± 13.7	57.2 ± 14.2	0.099	62.5 ± 9.1	64.6 ± 5.9	0.519

***Deceleration and acceleration capacities***				
AC (ms)	-5.5 ± 2.6	-4.0 ± 1.4	0.004	-4.2 ± 1.4	-3.7 ± 1.5	0.541
DC (ms)	5.1 ± 2.5	3.6 ± 1.4	0.003	3.9 ± 1.3	4.0 ± 1.6	0.908
***Stage of heart failure***				
IV	18 (40.0%)	8 (40.0%)	1.000	13 (43.3%)	3 (60.0%)	0.642
III	21 (46.7%)	10 (50.0%)	1.000	13 (43.3%)	0 (0.0%)	0.134
II	5 (11.1%)	2 (10.0%)	1.000	4 (13.3%)	2 (40.0%)	0.195
I	1 (2.2%)	0 (0%)	*/*	0 (0%)	0 (0%)	/

LAD (mm)	46.3 ± 7.3	53.6 ± 7.9	0.001	46.9 ± 7.3	51.8 ± 8.3	0.268
LVEDD (mm)	71.2 ± 9.3	71.1 ± 8.1	0.950	66.6 ± 7.0	75.0 ± 3.9	0.004
LVESD (mm)	59.4 ± 10.6	60.1 ± 10.0	0.814	57.6 ± 7.0	59.2 ± 18.0	0.857
LVEF (%)	30.4 ± 6.9	30.2 ± 7.8	0.920	30.6 ± 8.5	30.2 ± 5.1	0.888

*Continuous variables are presented as means ± standard deviations (SDs); differences between groups were compared using Student’s t-test. Categorical variables are presented as numbers (with percentages); differences between groups were compared using Fisher’s exact probability test. The stages of heart failure were those of the New York Heart Association (NYHA) Functional Classification. For abbreviations, please see Table 1*.

In females, only age was significantly associated with DC on the univariate linear logistic regression analysis with a *B* value of -0.04 (95% CI, -0.055 ∼-0.015; *P* = 0.001); a multivariable linear logistic regression analysis was thus not performed (Table 3).

In summary, age was an independent risk factor for DC in both male and female controls, and the LAD and LVEF were independent risk factors for DC in male DCM patients.

### Survival and Death of DCM Patients

To further explore whether the AC and DC differed between DCM patients who survived and died, the AC, DC, echocardiographic data, age, and stages of HF were compared between subgroups. As showed in Table 4, 65 males and 35 females with DCM were followed up; 20 males and 5 females died during the 60 months follow up. Of males, the average follow up time of survived and died groups were 29.2 ± 15.9 and 29.5 ± 14.6 months, respectively; Of females, the follow up time of survived and died groups were 33.2 ± 15.0 and 28.2 ± 15.4 months, respectively (Table 4). In males, the LAD and AC were significantly higher and the DC was significantly lower in those who died. In females, only the LVEDD differed significantly between those who died and survived, being significantly higher in the former cases (Table 4). In summary, in males, the absolute values of both AC and DC decreased and that of LAD increased significantly in those who died; in females, only the LVEDD was significantly higher in those who died.

### DCM Patients With a Higher AC or a Lower DC Are at Higher Risk of Cardiac Death

**FIGURE 3:**
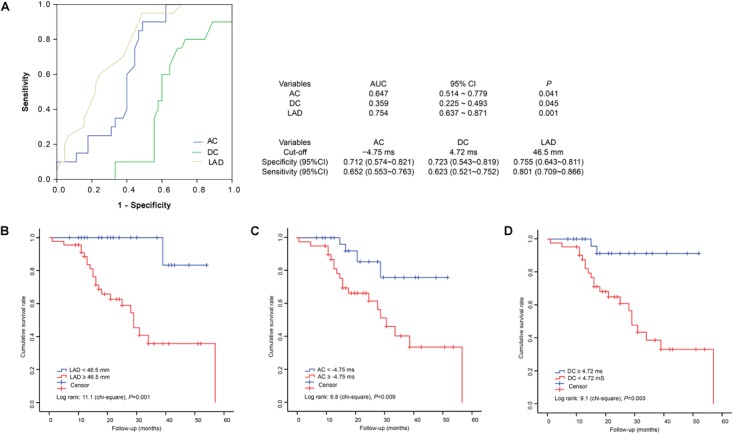
Receiver operating characteristic curves and Kaplan–Meier analysis of 60-month survival rates by LAD, AC, and DC status. ROC, receiver operating characteristic; AUC, areas under the ROC curves; 95%CI, 95% confidence interval. **(A)** ROC, AUC and cut-offs. **(B)** Cumulative survival rates of patients grouped by LAD status. **(C)** Cumulative survival rates of patients grouped by AC status. **(D)** Cumulative survival rates of patients grouped by DC status.

We found that AC, DC, and the LAD could predict the prognoses and survival of male DCM patients; we next calculated cumulative survival rates using appropriate cutoffs of these parameters. We first plotted ROC curves using the AC, DC, and LAD values of individual patients; the AUCs of AC, DC, and LAD were 0.647 (95%CI, 0.514 ∼ 0.779), 0.359 (95%CI, 0.225 ∼ 0.493), and 0.754 (0.637 ∼ 0.871), respectively (Figure 3A). The cutoffs for AC, DC, and the LAD were -4.75, 4.72, and 46.5 mm, respectively (Figure 3A). We next calculated cumulative survival rates using these cutoffs. During 60 months of follow-up, we counted deaths in subgroups formed using the cutoffs LAD ≥6.5 mm, AC ≥ –4.75 ms, and DC ≥ 4.72 ms; the numbers were 17, 16, and 15, respectively. Kaplan–Meier analysis revealed that DCM patients with greater LADs (log-rank Chi-square = 11.1, *P* = 0.001), a higher AC (log-rank Chi-square = 6.8, *P* = 0.009), and/or a lower DC (log-rank Chi-square = 9.1, *P* = 0.003) were at higher risk of cardiac death (Figure 3).

Our number of female patients was small and we could not identify any parameter predicting survival; we thus did not perform a Kaplan–Meier analysis of female DCM patients.

## Discussion

Adult patients with DCM generally have a 1-year mortality rate of 25–30% and only 50% survive to 5 years (Dec and Fuster, 1994; Weintraub et al., 2017). Currently, it is thought that a poorer prognosis is associated with an LVEF < 25%, right ventricular involvement, higher New York Heart Association functional class, and poor hemodynamic status (Keogh et al., 1990; Lewis et al., 1993; Dec and Fuster, 1994; Weintraub et al., 2017). We found that although all echocardiographic changes, including the LAD, LVEDD, LVESD, and LVEF, differed between both male and female controls and DCM patients; only the LAD and LVEDD were significantly greater in male and female DCM patients who died, respectively; no differences in any other echocardiographic parameter or HF stage were evident between those who died and survived. Cumulative survival analysis revealed that male patients with LADs ≥ 46.5 mm were at a significantly higher risk of cardiac death within 60 months of follow-up. We did not perform similar analyses in females because of the small number of deaths.

Heart rate is modulated by both the sympathetic and vagal nervous systems: the former accelerates and the latter decelerates the rate (Bauer et al., 2006). PRSA can quantify heart AC and DC under noisy conditions (such as when an external heart rhythm is in play) and when the signals are not stationary (e.g., in patients with arrhythmia) (Bauer et al., 2006; Kantelhardt et al., 2007). Based on these observations, the AC and DC should be better than the traditional measures of heart rate variability (HRV) (Hu et al., 2016; Zou et al., 2016). We evaluated associations between AC, DC, and DCM and found that the absolute values decreased significantly in DCM patients, which suggests parasympathetic activity was attenuated and sympathetic activity was enhanced in the heart of DCM. Second, in males, only age independently and positively associated with the AC increase in healthy controls; DCM changed this association pattern; the LVEF and LAD emerged independently (negatively and positively, respectively) associated with AC; suggesting that increased sympathetic activity associated with enlargement of the left atrium (LA) and LVEF reduction. Third, in males, age was the only factor negatively associated with DC in healthy controls; again, DCM changed this pattern; the LAD was negatively and the LVEF positively associated with DC; suggesting a decrease in parasympathetic activity associated with LA enlargement and a fall in the LVEF. Fourth, in females, only age was a risk factor for AC and DC; no echocardiographic change was associated with AC or DC. Although the number of female DCM patients was small, we suggest that this distinction was attributable principally to a between-gender difference. Fifth, Kaplan–Meier analysis revealed that male DCM patients with ACs ≥ –4.75 ms or DCs ≤ 4.72 ms were at higher risk of cardiac death within 60 months of follow-up. In summary, our data suggest that the structural and mechanical changes associated with DCM greatly affect cardiac electrophysiology, perhaps reflecting pathophysiological changes in terms of both sympathetic and parasympathetic regulation of cardiac action.

It has been very difficult to identify and quantify the roles played by the vagal and sympathetic nervous systems in terms of cardiac electrophysiology, not only because effective methods are lacking, but also because both types of nerves are complex in structure, containing mixtures of neural types (Chen et al., 2014). Few studies have focused on changes in cardiac regulation mediated by the autonomic nervous in DCM patients. One 1993 study used the log of the high-frequency power as an index of parasympathetic nervous activity and the log of the (low-frequency power/high-frequency power ratio) as an index of sympathetic nervous activity, and found that parasympathetic activity was attenuated and sympathetic activity was enhanced in DCM patients (Ajiki et al., 1993), which is consistent with our results. However, autonomic nervous system dysfunction in HF patients has been rather well studied (Florea and Cohn, 2014). An autonomic imbalance associated with an increase in sympathetic activity and withdrawal of vagal activity are representative neurophysiological characteristics of HF (Florea and Cohn, 2014). To the best of our knowledge, the non-invasive methods adopted in previous studies were HRV, baroreflex sensitivity, and heart rate turbulence analyses (Florea and Cohn, 2014). We earlier showed that AC and DC were independent risk factors for DCM, and that AC predicted HF exacerbation in DCM patients (Zou et al., 2016). In this report, we followed up these DCM patients for 5 years, and survival analysis revealed that male DCM patients with a higher AC or a lower DC were at higher risk of cardiac death. We also found that the LAD, but not the LVEF or LVESD, was an independent risk factor for both AC and DC, suggesting that LA enlargement may have a major effect on autonomic cardiac regulation. The intrinsic cardiac nerves are located principally in the atria (Chen et al., 2014), in line with the above suggestion.

Our results are consistent with a prior report focused on the associations between AC/DC and HF (Hu et al., 2016) at the following points: the DC was lower and the AC was higher in patients group; the LAD was positively and the LVEF was negatively associated with AC in patients; and the LAD was negatively and the LVEF was positively associated with DC in patients. The consistency of the results may be interpreted by DCM is one of the most common causes of HF. In addition, Hu et al showed that DC ≤ 4.55 ms and/or AC ≥-6.15 ms could discriminate males with HF with high performance (Hu et al., 2016); our results showed that male DCM patients with DC < 4.72 ms and/or AC > -4.75 ms were at higher risk of cardiac death over the 60 months of follow-up. Since our report is a longitudinal study and Hu et al.’s (2016) report is a cross-sectional one; and the dependent variables differed between these two studies; we can not read too much about the similarities and differences between these two studies.

We enrolled only 35 female DCM patients, and only 5 died during the 5 years of follow-up, so it was not possible to analyze female survival. This is a limitation of our report. Additionally, mutations in genes encoding cytoskeletal, sarcomere, and nuclear envelope proteins are known to account for up to 35% of all DCM cases (Weintraub et al., 2017); mutations in the LMNA, EMD, or SCN5A gene may trigger supraventricular arrhythmia and conduction disease (Lakdawala and Givertz, 2010). We did not explore the mutational status of our patients, so we cannot discuss how such mutations might affect AC and DC levels. However, PRSA excludes noise (such as the external rhythm of the heart) and non-stationary signals (e.g., arrhythmia) during AC and DC calculations, which reduces our concerns to some extent. Beta-adrenergic blockade inhibits sympathetic nervous activity and angiotensin-converting-enzyme (ACE) inhibitors may modulate the autonomic nervous system (Adamson et al., 1994; Task Force of the European Society of Cardiology the North American Society of Pacing Electrophysiology, 1996). However, others have found that neither ACE inhibitors nor beta-adrenergic blockade affected cardiac electrophysiology (Sanderson et al., 1999; Vaile et al., 2001; Hu et al., 2016). Because most of our patients were taking beta-blockers and ACE inhibitors, we cannot eliminate any possible effects of these drugs on HRV.

## Author Contributions

CZ and XY conceived and designed the work. YY, FW, HD, XH, BZ, and XL performed the experiments, and acquired, analyzed, and interpreted the data for the work. CZ and XY drafted the work and revised it critically for important intellectual content. YY, FW, CZ, HD, XH, BZ, XL, and XY approved the final version of the manuscript to be published.

## Conflict of Interest Statement

The authors declare that the research was conducted in the absence of any commercial or financial relationships that could be construed as a potential conflict of interest.
